# Casein Kinase 2α Ablation Confers Protection Against Metabolic Dysfunction‐Associated Steatotic Liver Disease: Role of FUN14 Domain Containing 1‐Dependent Regulation of Mitophagy and Ferroptosis

**DOI:** 10.1002/mco2.70277

**Published:** 2025-07-11

**Authors:** Ke He, Meixiao Zhan, Xuanming Luo, Ruibing Li, Ling Lin, Jie Lin, Liheng Li, Hongdong Chen, Gary D. Lopaschuk, Hao Zhou, Fei Liu, Jun Ren

**Affiliations:** ^1^ Minimally Invasive Tumor Therapies Center Guangdong Second Provincial General Hospital Guangzhou China; ^2^ Guangzhou First People's Hospital The Second Affiliated Hospital School of Medicine South China University of Technology Guangzhou China; ^3^ Guangdong Provincial Key Laboratory of Tumor Interventional Diagnosis and Treatment Zhuhai People's Hospital (Zhuhai Hospital Affiliated with Jinan University) Zhuhai China; ^4^ Department of Biliary Tract Surgery Zhongshan Hospital Fudan University Shanghai China; ^5^ Department of General Surgery Shanghai Xuhui Central Hospital Fudan University Shanghai China; ^6^ Department of Clinical Laboratory Medicine The First Medical Centre Medical School of Chinese People's Liberation Army Beijing China; ^7^ Department of Cardiology Shanghai Institute of Cardiovascular Diseases Zhongshan Hospital Fudan University Shanghai China; ^8^ National Clinical Research Center for Interventional Medicine Zhongshan Hospital Fudan University Shanghai China; ^9^ Cardiovascular Research Centre University of Alberta Edmonton Canada; ^10^ Senior Department of Cardiology The Sixth Medical Center of People's Liberation Army General Hospital Beijing China

**Keywords:** casein kinase 2α, ferroptosis, FUN14 domain containing 1, MASLD, steatosis

## Abstract

Mitochondrial dyshomeostasis provokes the onset of metabolic dysfunction‐associated steatotic liver disease (MASLD) although its precise involvement in particular mitophagy in MASLD remains elusive. This work evaluated the role of casein kinase 2α (CK2α) and FUNDC1 in high‐fat diet (HFD)‐evoked MASLD. WT and CK2α deletion (CK2α*^‐/‐^*) mice were subjected to low fat or HFD for 20 weeks. Global metabolism, AST, ALT, cholesterol, triglycerides, hepatic steatosis, fibrosis, inflammation, mitochondrial injury, mitophagy and ferroptosis were examined. Bioinformatics analysis enriched mitochondria‐related pathways in MASLD. Hepatic CK2α and FUNDC1 were upregulated and downregulated, respectively, in MASLD patients and HFD‐fed mice. HFD led to adiposity, hepatomegaly, hepatic steatosis, fibrosis, inflammation, ferroptosis, mitochondrial injury, elevated hepatic tissue Fe^2+^, FAS, CHREBP, SREBP1, PGC1α, PPARα, PPARγ, SCD1, PEPCK, G6Pase, and DGAT1 as well as downregulated FUNDC1, GPx4, SLC7A11 and NCOA4, the effects (except for NCOA4) were nullified by CK2α deletion. FUNDC1 deletion nullified CK2α deletion‐evoked benefit on hepatic ferroptosis and lipid enzymes. In vitro study using palmitic acid indicated an obligatory role for CK2α, FUNDC1 and ferroptosis in hepatocyte steatosis. Collectively, our results demonstrated that CK2α activation by HFD serves as a trigger for mitochondrial damage, hepatic injury, and pathogenesis of MASLD through FUNDC1 disruption and ferroptosis.

## Graphical abstract

Schematic graph exhibiting possible mechanism(s) for the role of CK2α and FUNDC1 in high fat diet‐induced hepatic steatosis. Long‐term high fat diet insult dampens FUNDC1‐mediated mitophagy through upregulation of CK2α, prompting disturbed lipid metabolism and lipid accumulation due to compromised mitophagy. Lipotoxicity subsequently promotes lipotoxic ferroptosis, imperiling hepatic steatosis.

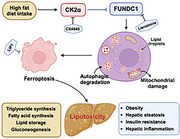

## 1 Introduction

Metabolic dysfunction‐associated steatotic liver disease (MASLD) is a serious liver disorder commonly associated with insulin resistance, visceral adiposity, and metabolic syndrome, all of which contribute to hepatic fat accumulation and progression to metabolic‐associated steatotic hepatitis (MASH) [1, 2]. This progression is characterized by liver fibrosis, inflammation, and a heightened risk of cirrhosis and cancer [3, 4]. The hallmark of MASLD is fat accumulation within hepatocytes, leading to pronounced lipotoxicity, oxidative stress, mitochondrial dysfunction, and cell death through complex signaling pathways [5, 6, 7]. In addition, MASLD is linked to dyslipidemia, cardiovascular comorbidities, and alterations in gut microbiota, while conditions like sleep apnea and chronic kidney disease may further exacerbate metabolic complications [7, 8]. Key culprit factors such as inflammation, endoplasmic reticulum (ER) stress, lipotoxicity, and disrupted autophagy have been depicted to contribute to the progression of MASLD [6, 9, 10, 11]. However, the precise mechanisms underlying MASLD remain unclear.

Recent studies have emphasized the critical role of mitophagy, a mitochondria‐selective autophagic process essential for maintaining mitochondrial quality, function, and insulin sensitivity, in the etiology of MASLD [12]. Evidence suggests that enhancing mitophagy in metabolic disorders, such as diabetes mellitus and obesity, can mitigate mitochondrial damage, oxidative stress, and apoptosis, while improving mitochondrial ATP production and function [13]. Notably, adequate mitophagy in response to high glucose or fatty acid exposure protects against cell death by preserving mitochondrial homeostasis [14], underscoring its importance as a protective mechanism for hepatocyte survival under conditions of metabolic excess.

To date, three components namely Parkin, FUN14 domain containing 1 (FUNDC1), and BCL2/adenovirus E1B 19 kDa protein‐interacting protein‐3 (BNIP3) are perceived as central mitophagy elements [14]. Earlier studies suggest that BNIP3‐related mitophagy plays a protective role in preventing liver cancer metastasis and MASLD [15, 16]. In addition, other research underscores the pivotal role of Parkin in safeguarding against MASLD [17]. Findings from our group and others have highlighted the crucial function of FUNDC1‐related mitophagy in mitigating ischemia‐reperfusion‐induced organ damage [18, 19, 20, 21, 22]. At the molecular level, FUNDC1‐mediated mitophagy is inhibited via post‐transcriptional phosphorylation at Ser [13]. This phosphorylation creates a steric hindrance that impedes the interaction between FUNDC1 and LC3II, effectively halting mitophagy [23]. Further evidence has indicated that FUNDC1 suppresses the chemical carcinogen diethylnitrosamine‐induced hepatic cancer [24, 25]. However, a direct link between disturbed FUNDC1 and the development of MASLD remains unexplored.

Casein kinase 2α (CK2α) is a constitutive Ser/Thr kinase that inactivates FUNDC1 by phosphorylating it at Ser [13] [26]. Our earlier work demonstrated the critical role of elevated CK2α in the etiology of cardiac ischemia‐reperfusion injury, where it phosphorylates and inactivates FUNDC1, leading to compromised mitochondrial quality [22]. These findings suggest that CK2α serves as an essential culprit factor in maintaining mitochondrial homeostasis. Interestingly, increased CK2α levels have been observed in the livers of patients with MASLD and hepatic carcinoma [27, 28]. Emerging evidence indicates that CK2α functions as an independent pathological driver in the development of MASLD [27, 28], although the mechanisms underlying CK2α‐induced hepatic regulation remain poorly understood. Therefore, the present work aims to investigate the role of CK2α in mitochondrial abnormalities and MASLD. Given the close correlation between compromised mitophagy (including FUNDC1) and MASLD pathology [16, 17, 29], the possible involvement of FUNDC1‐mediated mitophagy was scrutinized in the realm of CK2α‐evoked hepatic regulation, if any.

## 2 Results

### 2.1 Bioinformatic Analysis of Mitochondrial Dysfunction in the Etiology of MASLD

**FIGURE 1:**
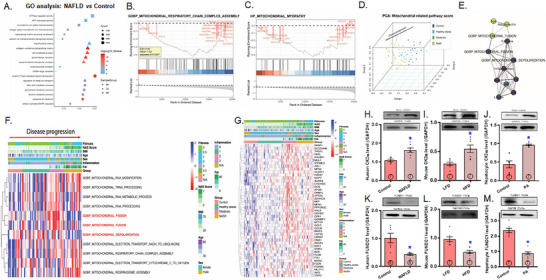
Bioinformatic analysis of mitochondrial function, expression of CK2α and FUNDC1 in the pathogenesis of metabolic dysfunction‐associated steatotic liver disease (MASLD) in human, mice, and primary mouse hepatocytes. (A–G): Bioinformatic analysis of mitochondrial function; A: GO analysis of differentially expressed genes (DEGs) among control and MASLD human liver samples; B,C: Mitochondrial pathways significantly enriched in GSEA analysis; D: PCA analysis of pathways quantified by GSVA (normalized quantification pathway score); E: Interaction between GSVA score of mitochondria related pathway and clinical features in partial correlation analysis; F: Heat map exhibiting the relationship between multifactorial clinical features and GSVA score of mitochondrial pathway; G: Heatmap displaying relationship between clinical features and enriched mitochondrial genes; H–J: Expression of CK2α in livers from MASLD patients, high‐fat diet (HFD)‐fed mice (60% fat diet for 20 weeks) and palmitic acid (0.5 mM for 72 h)‐challenged primary mouse hepatocytes; K–M: Expression of mitophagy protein FUNDC1 in livers from MASLD patients, HFD‐fed mice and palmitic acid (0.5 mM for 72 h)‐challenged primary mouse hepatocytes. Mean ± SEM, sample size (*n*) is indicated within the closed circle on each bar, *p < 0.05 vs. respective control or low‐fat diet (LFD) group.

To discern the possible contributing factors for mitochondrial dysfunction in MASLD, bioinformatic analysis was conducted to identify key molecular functions (MFs), cellular components (CC), and biological processes (BP) involved in this pathology. MF analysis from the Gene Ontology (GO) study highlights involvement in BP such as ATP hydrolysis (adjusted *p* value = 5.71E‐05), voltage‐gated monoatomic ion channel activity (adjusted *p* value = 3.79E‐08), and GTPase regulatory function (adjusted *p* value = 5.74E‐06) in the etiology of MASLD. Significantly enriched CC terms include mitochondrial matrix (adjusted *p* value = 1.62E‐04), cell‐substrate junction (adjusted *p* value = 1.44E‐15), and collagen‐containing extracellular matrix (adjusted *p* value = 2.26E‐18). In addition, the BP terms reveal enrichment in pathways such as small GTPase‐mediated signal transduction (adjusted *p* value = 4.44E‐17), response to metal ions (adjusted *p* value = 9.15E‐14), and alcohol metabolic process (adjusted *p* value = 2.55E‐08) (Figure 1A). The GO analysis results indicate that mitochondrial dysfunction and aberrant energy metabolism are the primary regulatory pathways involved in the etiology of MASLD. To investigate further, Gene Set Enrichment Analysis (GSEA) analysis was conducted on mitochondrial pathway‐related genes. Differentially expressed genes (DEGs) related to mitochondria were found to be downregulated in pathways such as GOBP_MITOCHONDRIAL_RESPIRATORY_CHAIN_COMPLEX_ASSEMBLY [ES = −0.42, normalized enrichment scores (NES) = −1.52, B‐H adjusted *p* values = 0.0057] and HP_MITOCHONDRIAL_MYOPATHY [ES = −0.43, normalized enrichment scores (NES) = −1.46, B‐H adjusted *p* values = 0.026] (Figure 1B,C). To further explore the relationship between mitochondrial pathways and MASLD, all mitochondria‐related pathways were scored using Gene Set Variation Analysis (GSVA). A three‐dimensional principal component analysis (PCA) analysis demonstrated that mitochondrial pathways were consistently associated with MASLD pathology to contribute to the etiology of MASLD (Figure 1D). Moreover, the relationship among mitochondrial pathways, MASLD progression, and clinical features was visually evaluated. The results highlight the crucial roles of mitochondrial‐related processes, such as GOBP_MITOCHONDRIAL_FISSION, GOBP_MITOCHONDRIAL_FUSION, and GOBP_MITOCHONDRIAL_ DEPOLARIZATION in the development of MASLD. These processes were found to correlate with clinical features, including liver fibrosis, nonalcoholic fatty liver disease activity score (NAS) score, inflammation, body mass index (BMI), and body fat (Figure 1E).

Furthermore, we conducted partial correlation analysis and created an interaction graph for mitochondrial‐related pathways, including GOBP_MITOCHONDRIAL_FISSION, GOBP_MITOCHONDRIAL_FUSION, GOBP_MITOCHONDRIAL_DEPOLARIZATION, all of which play critical roles in the development of MASLD, alongside various clinical features (Figure 1F). These results reveal strong associations between these three pathways and clinical features such as liver fibrosis, NAS score, inflammation, and body fat. To further investigate the roles of these pathways in MASLD progression, we generated a heat map to illustrate the interplay between the genes involved in the aforementioned pathways and key clinical features, including fibrosis, NAS score, inflammation, BMI, and body fat (Figure 1G). The findings demonstrate a profound decline in the levels of these genes in MASLD, with distinct variations corresponding to levels of fibrosis, NAS score, inflammation, BMI, and body fat.

### 2.2 Changes of CK2α and FUNDC1 in Livers From MASLD Patients and High‐Fat Diet (HFD)‐Fed Mice, Biometric Profiles in Mice With CK2α and/or FUNDC1 Knockout Consuming HFD

**FIGURE 2:**
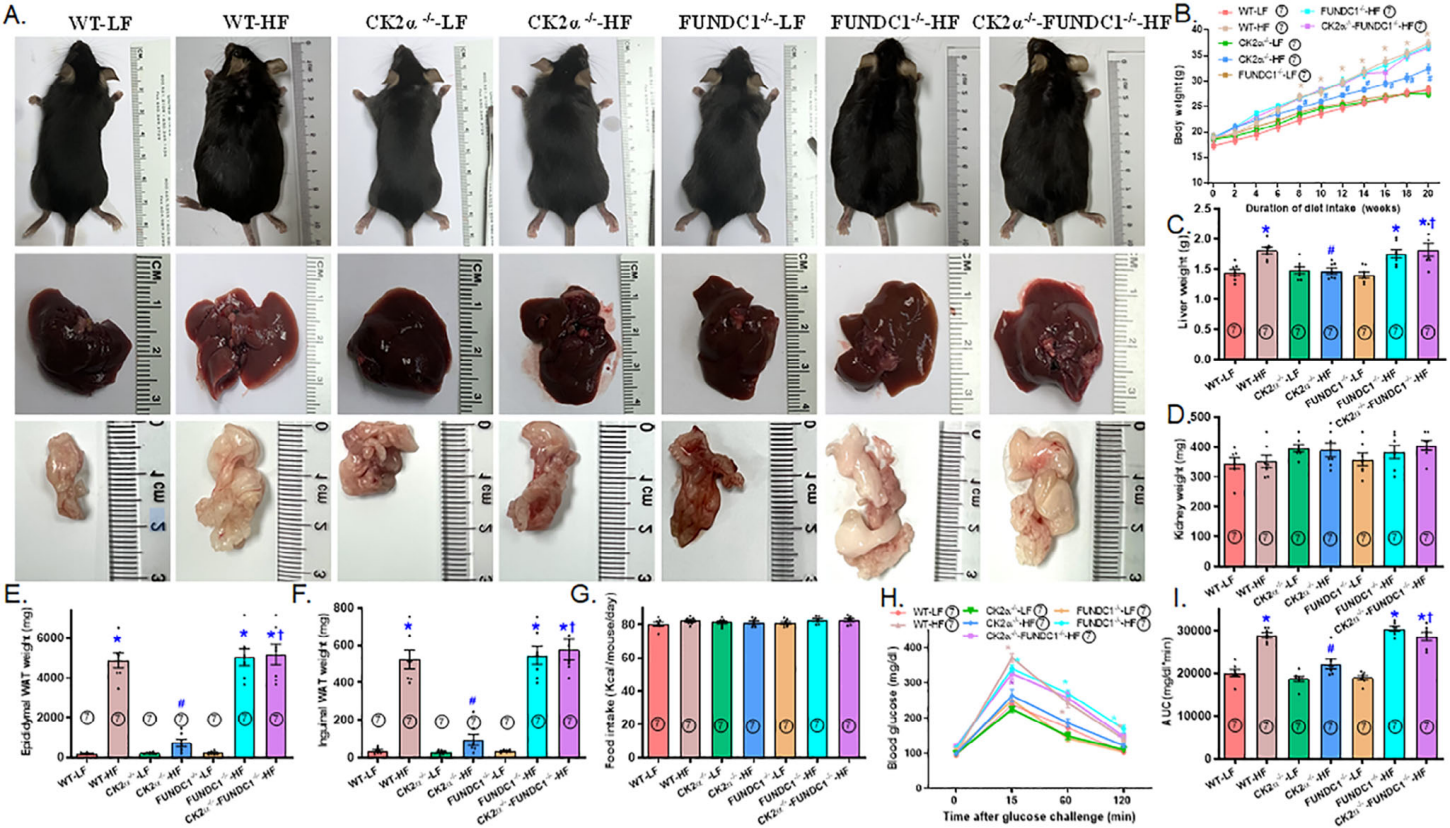
Biometric properties in 5‐week–old WT, CK2α knockout (CK2α–^/–^), FUNDC1 knockout (FUNDC1–^/–^) and CK2α‐FUNDC1 double knockout (CK2α–^/—^FUNDC1–^/–^) mice placed on a 60% high fat (HF) or nutritionally matched low fat (LF) diet for 20 weeks. A: Representative photographs of mice (prone and supine positions), liver and white adipose tissues; B: Weekly trajectory of body weight in mice from various groups; C: Liver weight; D: Kidney weight; E: Inguinal white adipose tissue (WAT) weight; F: Epididymal WAT weight; G: Intraperitoneal glucose tolerance test (IPGTT); H: Area under curve (AUC) for IPGTT; and I: Food intake (per mouse daily). Mean ± SEM, sample size (*n*) is indicated within the closed circle on each bar, *p < 0.05 vs. WT mice, #*p* < 0.05 vs. WT‐HF mice, †*p* < 0.05 vs. CK2α–^/—^HF mice.

To discern the interplay between CK2α and FUNDC1 in the context of MASLD, human and mouse liver samples were employed to elucidate their roles in metabolic regulation and disease progression. Evaluation of liver tissues from patients with MASLD and HFD‐fed mice revealed an upregulation of the proinflammatory marker CK2α and downregulation of the mitophagy receptor FUNDC1. Similarly, in primary hepatocytes isolated from C57BL/6 mice challenged with palmitic acid (PA, 100 µM for 24 h) in vitro, CK2α levels were elevated, while FUNDC1 expression was downregulated, (Figure 1H–M). To further discern the roles of CK2α and FUNDC1 in the etiology of MASLD, CK2α^‐/‐^, FUNDC1^‐/‐^, CK2α^‐/–^ FUNDC1^‐/‐^ double knockout and WT mice were offered either a low‐fat diet (LFD) or HFD diet for 20 weeks. HFD intake led to an evident rise in body and liver (but not kidney) weight, which was effectively reversed by CK2α ablation. Similarly, HFD‐induced adiposity, as evidenced by increased epididymal and inguinal white adipose tissue (WAT) weights, was also mitigated by CK2α deletion. Glucose sensitivity assessments demonstrated that HFD consumption resulted in glucose intolerance [delayed return of blood glucose to baseline and higher area under the curve (AUC)] despite normal basal blood glucose levels. These adverse effects were alleviated by CK2α deletion. CK2α ablation alone failed to exert any discernable response on body or organ weights, nor on glucose tolerance under LFD conditions. Intriguingly, ablation of the mitophagy receptor FUNDC1 negated the beneficial effects of CK2α deletion on body weight, liver weight, WAT (epididymal and inguinal), and glucose intolerance under HFD challenge, while FUNDC1 deletion alone did not produce any significant effects (Figure 2A–I).

### 2.3 Whole Body Metabolism, Hepatic Steatosis, and Liver Function

**FIGURE 3:**
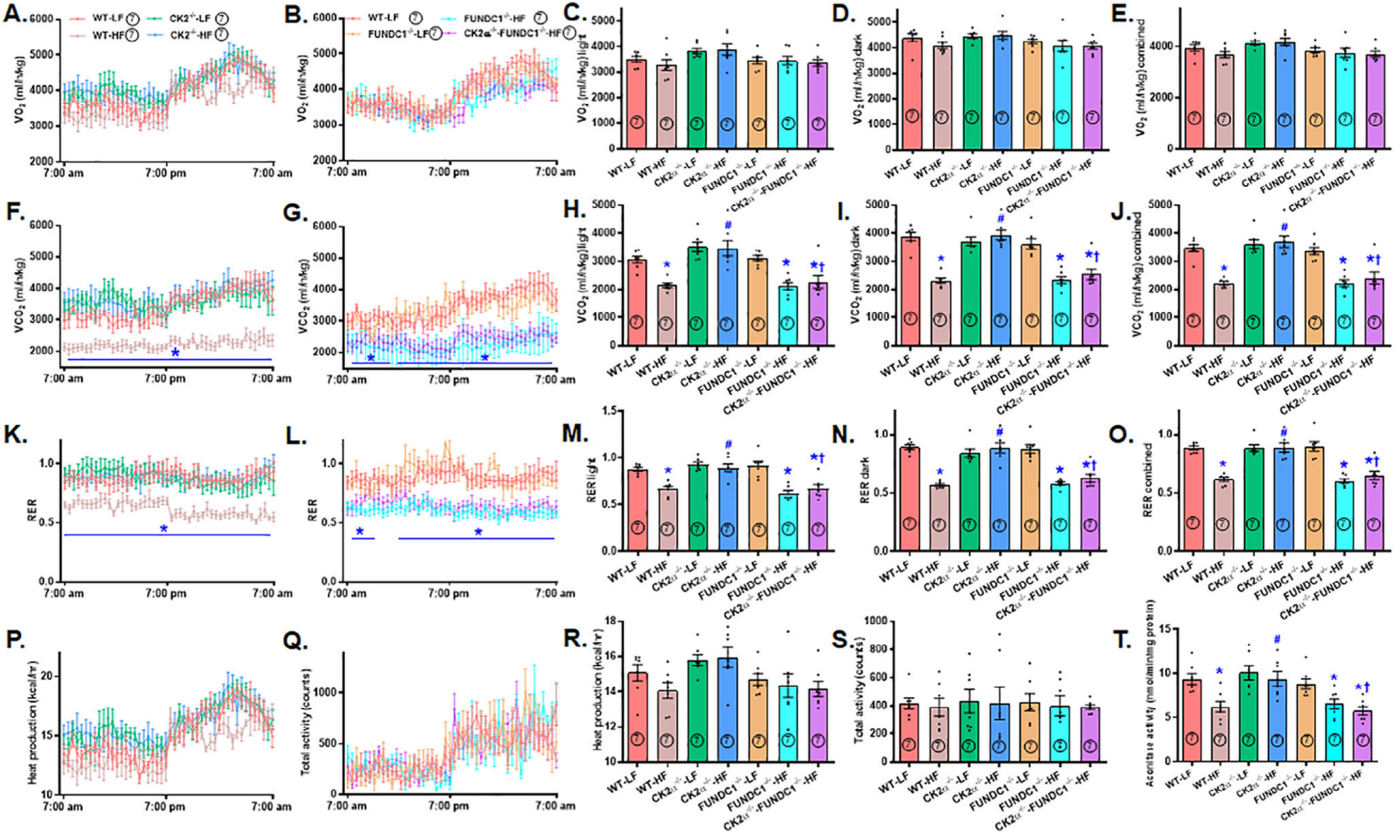
Metabolic properties (24‐hr cycle) of 5‐week–old WT, CK2α knockout (CK2α–^/–^), FUNDC1 knockout (FUNDC1–^/–^) and CK2α‐FUNDC1 double knockout (CK2α–^/—^FUNDC1–^/–^) mice placed on a 60% high fat (HF) or nutritionally matched low fat (LF) diet for 20 weeks. A: O_2_ consumption in WT and CK2α^‐/‐^mice; B: O_2_ consumption in FUNDC1^‐/‐^ and CK2α^‐/–^FUNDC1^‐/‐^ mice; C: Pooled O_2_ consumption (light); D: Pooled O_2_ consumption (dark); E: Pooled O_2_ consumption (combined); F: CO_2_ production in WT and CK2α^‐/‐^ mice; G: CO_2_ production in FUNDC1^‐/‐^ and CK2α^‐/–^FUNDC1^‐/‐^ mice; H: Pooled CO_2_ production (light); I: Pooled CO_2_ production (dark); J: Pooled CO_2_ production (combined); K: Respiratory exchange ratio (RER) in WT and CK2α^‐/‐^ mice; L: RER ratio in FUNDC1^‐/‐^ and CK2α^‐/–^FUNDC1^‐/‐^ mice; M: Pooled data of RER (light); N: Pooled data of RER (dark); O: Pooled data of RER (combined); P: Heat production; Q: Total physical activity; R: Pooled data of heat production (24 h); S: Pooled data of total physical activity; and T: Aconitase activity. Mean ± SEM, sample size (n) is indicated within the closed circle on each bar. *p < 0.05 vs. WT mice, #*p* < 0.05 vs. WT‐HF mice, †*p* < 0.05 vs. CK2α–^/—^HF mice.

To investigate potential scenarios underlying CK2α‐ and FUNDC1‐mediated biometric and metabolic response, a CLASM system was utilized to assess whole‐body metabolism across a 24‐h period. The results demonstrated that HFD significantly reduced CO_2_ production and respiratory exchange ratio (RER) while having no effect on O_2_ intake, heat generation, or physical activity. These metabolic disturbances were mitigated by CK2α ablation, though CK2α ablation had no impact under LFD conditions. Interestingly, the protective effects of CK2α ablation against HFD‐induced metabolic changes were abolished by the removal of FUNDC1, with FUNDC1 deletion exerting minimal effects under LFD intake (Figure 3A–S). Further evaluation of mitochondrial function using an aconitase assay revealed that HFD significantly decreased liver aconitase activity, a marker of mitochondrial performance. This decline was reversed by CK2α deletion. However, the beneficial effect of CK2α ablation on aconitase activity under HFD conditions was nullified by FUNDC1 deletion, while FUNDC1 knockout alone had little effect under LFD conditions (Figure 3T).

**FIGURE 4:**
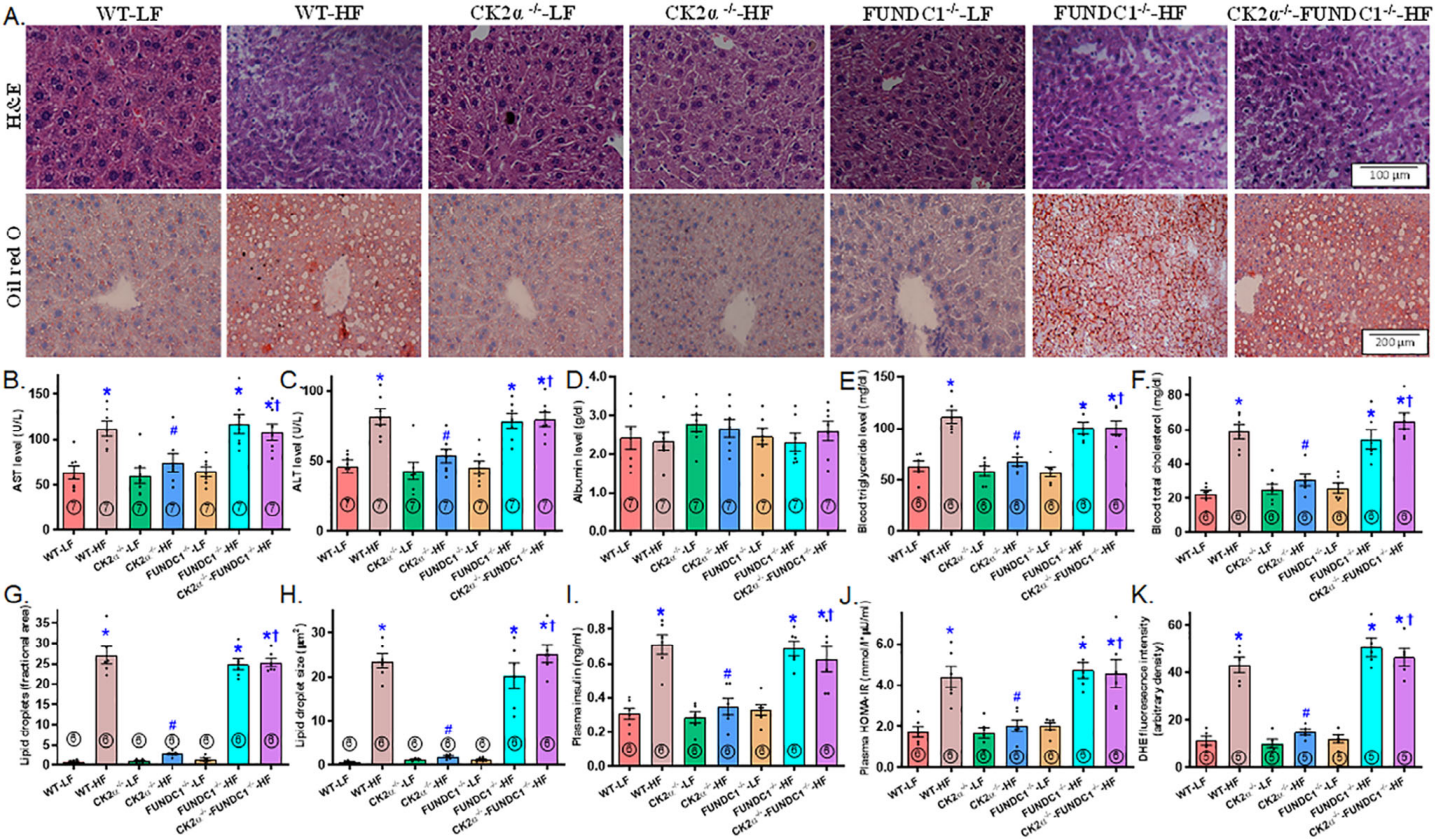
Hematoxylin and eosin (HE) staining and oil red O staining of liver sections of WT, CK2α knockout (CK2α–^/–^), FUNDC1 knockout (FUNDC1–^/–^) and CK2α‐FUNDC1 double knockout (CK2α–^/—^FUNDC1–^/–^) mice placed on a 60% high fat (HF) or nutritionally matched low fat (LF) diet for 20 weeks. A: Representative HE and oil red O staining of liver sections from mice fed LF or HF diet for 20 weeks; B: Serum AST levels; C: Serum ALT levels; D: Serum albumin levels; E: Blood triglyceride levels; F: Blood total cholesterol level; G: Lipid droplet area (% total field) from oil red O staining; H: Lipid droplet size (oil red O staining); I: Plasma insulin levels; J: Plasma HOMA‐IR index; and K: Liver DHE levels. Means ± SEM, sample size (*n*) is indicated within the closed circle on each bar. *p < 0.05 vs. WT mice, #*p* < 0.05 vs. WT‐HF mice, †*p* < 0.05 vs. CK2α–^/—^HF mice.

To assess HFD‐induced liver steatosis and tissue injury, we evaluated hepatic architecture, aspartate aminotransferase (AST), alanine aminotransferase (ALT), triglycerides, and cholesterol levels. In contrast to the classical lobular architecture observed in the LFD group, HFD consumption caused microvesicular fat buildup, particularly in the centrilobular zone, accompanied by hepatocyte ballooning and cytoplasmic rarefaction (Figure 4A). HFD intake also led to significant liver steatosis (visualized by Oil Red O staining, Figure 4A), elevated levels of AST, ALT, triglycerides, and cholesterol, as well as increased hepatic lipid droplet formation (both in number and size), plasma insulin and the homeostasis model assessment of insulin resistance (HOMA‐IR) index, while plasma albumin levels remain unchanged (Figure 4B–J). Although CK2α ablation exerted little response in LFD‐fed mice, it markedly alleviated HFD‐induced changes, including hepatic architectural alterations, liver steatosis, blood profiles indicative of liver injury, lipid droplet formation, plasm insulin levels, and the HOMA‐IR index. However, similar to its effects on global biometrics and metabolism, FUNDC1 deletion nullified the protective benefits of CK2α knockout against HFD‐induced liver changes. FUNDC1 deletion alone did not significantly affect liver morphology, blood parameters, lipid droplet formation, or hyperinsulinemia under HFD conditions (Figure 4A–J). In addition, elevated oxidative stress in HFD‐fed mice, as indicated by DHE staining, was effectively mitigated by CK2α knockout. While FUNDC1 deletion did not independently influence oxidative stress, it abolished the benefits provided by CK2α knockout under HFD conditions (Figure 4K).

### 2.4 Hepatic Fibrosis, Ultrastructure, Terminal Deoxynucleotidyl Transferase‐Mediated dUTP‐Biotin Nick End Labeling Assay (TUNEL) Apoptosis, and Adipocyte Morphology

**FIGURE 5:**
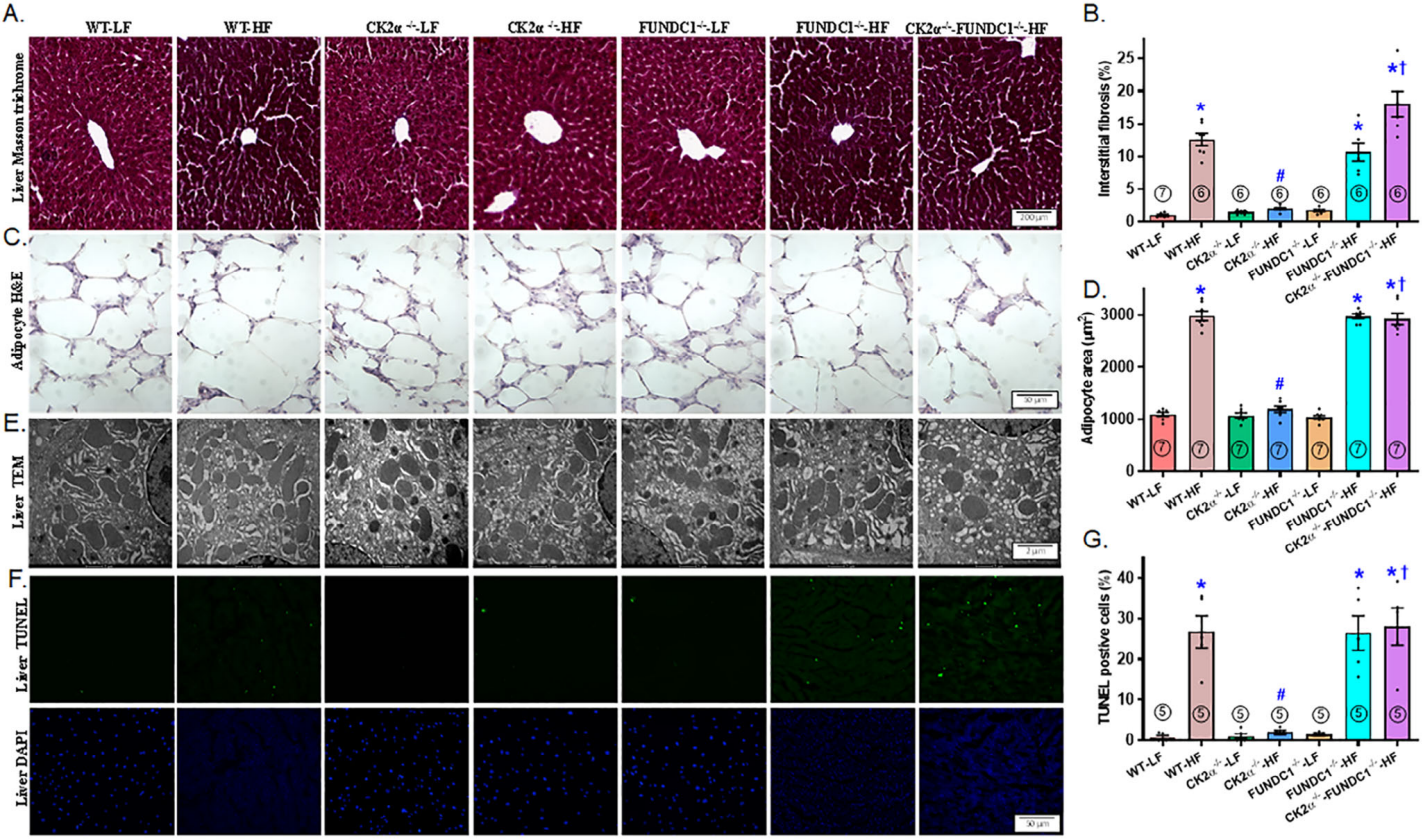
Hepatic fibrosis, ultrastructure, and apoptosis along with adipocyte size in WT, CK2α knockout (CK2α–^/–^), FUNDC1 knockout (FUNDC1–^/–^) and CK2α‐FUNDC1 double knockout (CK2α–^/—^FUNDC1–^/–^) mice placed on a 60% high fat (HF) or nutritionally matched low fat (LF) diet for 20 weeks. A: Representative Masson trichrome staining of liver sections from mice fed LF or HF diet for 20 weeks; B: Pooled hepatic fibrosis; C: Representative adipocyte HE staining from mice fed LF or HF diet for 20 weeks; D: Pooled adipocyte area; E: Representative TEM images exhibiting mitochondrial injury. Damaged mitochondria are shown by disorganization and reduced crista, and signs of vacuolation; F: Representative TUNEL staining of liver sections from mice fed LF or HF diet for 20 weeks; and G: Pooled TUNEL staining manifested as percent TUNEL‐positive cells. Means ± SEM, sample size (*n*) is indicated within the closed circle on each bar. *p < 0.05 vs. WT mice, #*p* < 0.05 vs. WT‐HF mice, †*p* < 0.05 vs. CK2α–^/—^HF mice.

Assessment of interstitial fibrosis, TEM ultrastructure, and apoptosis revealed evident hepatic interstitial fibrosis, disrupted ultrastructure (characterized by swelling, disorganization, and a reduction or loss of mitochondrial cristae), and increased TUNEL‐positive apoptotic cells following HFD intake. These pathological changes were effectively mitigated by CK2α knockout. However, the protective effects of CK2α deletion against HFD‐evoked hepatic interstitial fibrosis, ultrastructural aberrations, and apoptosis were nullified by FUNDC1 deletion, while FUNDC1 knockout alone exerted minimal effects. Given the pronounced changes in lipid profile and lipid droplets, we also examined adipocyte morphology. Our findings showed that HFD consumption robustly enlarged adipocyte size, a response that was reversed by CK2α deletion, with no noticeable effects in LFD‐fed mice. Consistent with earlier observations, the deletion of FUNDC1 negated the protective effects of CK2α ablation against HFD‐induced adipocyte hypertrophy, while FUNDC1 knockout alone had little impact under LFD conditions (Figure 5A–G).

### 2.5 Lipid Metabolism in the Liver

**FIGURE 6:**
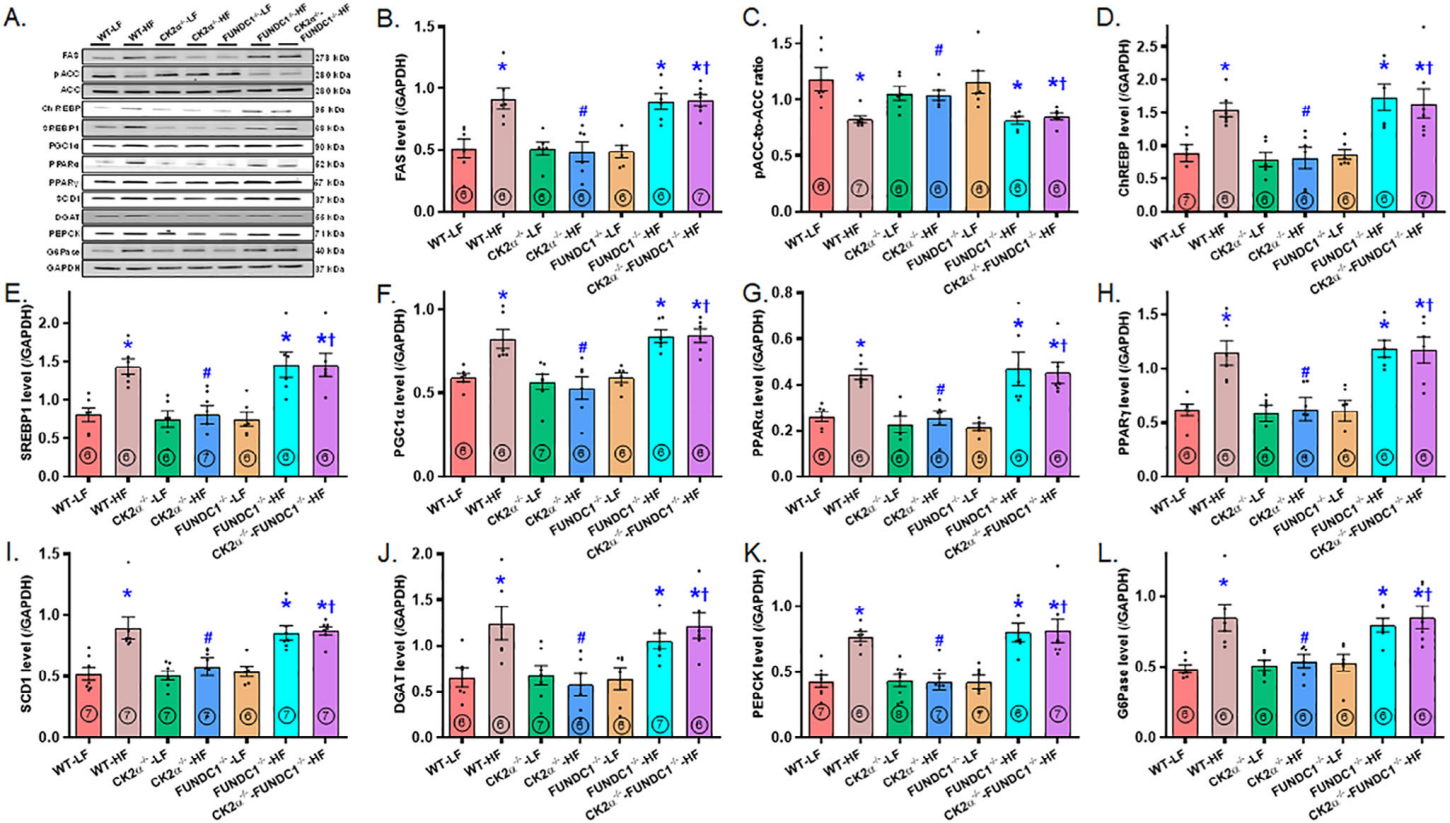
Levels of lipid regulating enzymes in livers from WT, CK2α knockout (CK2α–^/–^), FUNDC1 knockout (FUNDC1–^/–^) and CK2α‐FUNDC1 double knockout (CK2α–^/—^FUNDC1–^/–^) mice placed on a 60% high fat (HF) or nutritionally matched low fat (LF) diet for 20 weeks. A: Representative immunoblots depicting FAS, ACC (pan and phosphorylated), ChREBP, SREBP1, PGC1α, PPARα, PPARγ, SCD1, DGAT, PEPCK and G6Pase (GAPDH as loading control); B: FAS levels; C: pACC‐to‐ACC ratio; D: ChREBP levels; E: SREBP1 levels; F: PGC1α levels; G: PPARα levels; H: PPARγ levels; I: SCD1 levels; J: DGAT levels; K: PEPCK levels; and L: G6Pase levels. Mean ± SEM, sample size (*n*) is indicated within the closed circle on each bar. *p < 0.05 vs. WT mice, #*p* < 0.05 vs. WT‐HF mice, †*p* < 0.05 vs. CK2α–^/—^HF mice.

To investigate the effect of CK2α knockout on HFD‐provoked hepatic lipid buildup, we analyzed the expression of enzymes involved in lipid synthesis, uptake, and transport using immunoblotting. The results revealed a significant upregulation of key enzymes and transcriptional factors associated with lipid metabolism following HFD intake. These included fatty acid synthase (FAS), lipogenic transcription factors sterol regulatory element‐binding protein 1 (SREBP1) and carbohydrate‐responsive element‐binding protein (CHREBP), metabolic regulators peroxisome proliferator‐activated receptor gamma coactivator 1‐alpha (PGC1α), and peroxisome proliferator‐activated receptor α (PPARα); the lipid storage‐associated factor peroxisome proliferator‐activated receptor γ (PPARγ), the unsaturated fatty acid synthesis enzyme stearoyl–CoA desaturase 1 (SCD1), gluconeogenic enzymes glucose 6‐phosphatase (G6Pase) and phosphoenolpyruvate carboxykinase (PEPCK), and triglyceride synthesis enzyme diglyceride O‐acyltransferase 1 (DGAT1). These elevations were effectively mitigated by CK2α ablation, while CK2α deletion alone had minimal impact under LFD conditions. Intriguingly, the beneficial effects of CK2α ablation on lipid metabolic enzyme expression in the context of HFD intake were negated by FUNDC1 deletion. FUNDC1 knockout alone did not significantly alter the levels of these lipid metabolism‐related enzymes under LFD conditions (Figure 6A–L).

### 2.6 Hepatic Inflammation, Apoptosis, Mitophagy, and Ferroptosis

To discern the possible involvement of inflammation, mitophagy, and cell death pathways in the CK2α knockout‐elicited protection against HFD‐induced hepatic steatosis, we examined the expression of key protein markers related to these domains. HFD intake significantly upregulated the levels of CK2α, tumor necrosis factor‐α (TNFα), interleukin 1β (IL1β), caspase 3, the mitophagy marker translocase of outer mitochondrial membrane 20 (TOM20) (which indicates dampened mitophagy) and hepatic tissue iron (Fe^2+^) load, while downregulating the levels of FUNDC1, ferroptosis markers glutathione peroxidase 4 (GPx4), solute carrier family 7, member 11 (SLC7A11), and nuclear receptor coactivator 4 (NCOA4). These changes were reversed or masked by CK2α ablation, except for the ferritinophagy marker NCOA4. No notable changes were observed in the LFD group, aside from the expected decrease in CK2α levels. As observed in earlier findings, FUNDC1 deletion nullified (or masked) the beneficia; effects of CK2α ablation against HFD‐induced alterations, with minimal effects observed by itself under LFD conditions (Figure S1A–L, for Supporting Information).

### 2.7 Role of Mitophagy and Ferroptosis in Palmitic Acid‐Instigated Changes in Lipid Accumulation

**FIGURE 7:**
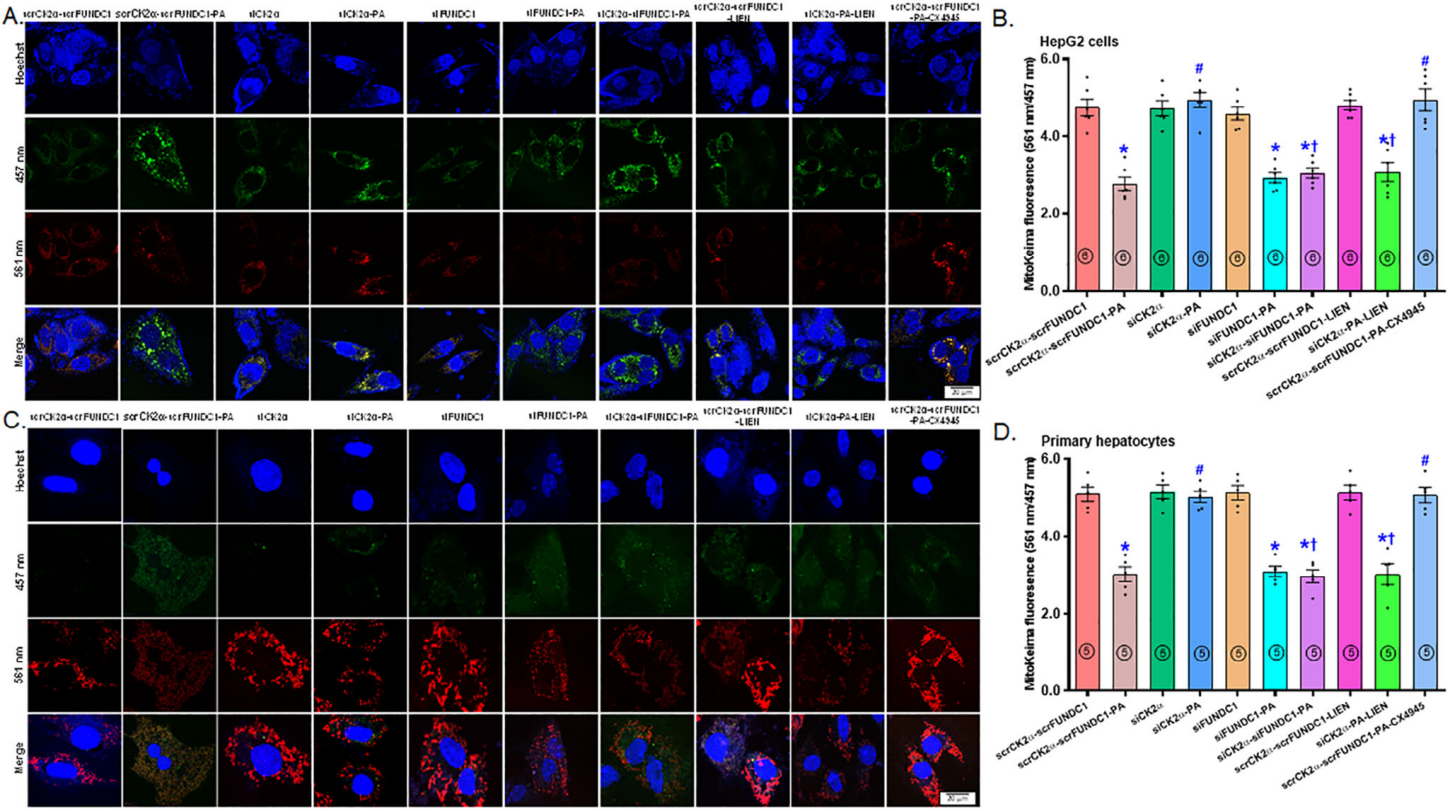
Effect of CK2α and FUNDC1 knockdown as well as CK2α selective inhibitor CX4945 (Silmitasertib) on palmitic acid (PA)‐induced changes in mitoKeima mitophagy in HepG2 cells and primary murine hepatocytes. HepG2 cells or primary murine hepatocytes were transfected with siCK2α or siFUNDC1 prior to exposure to PA (0.5 mM) in the presence or absence of the ferroptosis inducer erastin (ERA, 20 µM), the ferroptosis inhibitor LIP1 (200 nM) or the mitophagy inhibitor liensinine (LIEN, 20 µM) for 72 h. A subset of PA‐treated HepG2 cells or primary hepatocytes were incubated with the CK2α selective inhibitor CX4945 (10 µM). Scramble (scr) RNA serves as the negative control. A: Representative mitoKeima images depicting mitophagy from indicated HepG2 groups; B: Quantification of mitoKeima staining exhibiting HepG2 cellular mitophagy levels; C: Representative mitoKeima images depicting mitophagy from indicated primary murine hepatocyte groups; and D: Quantification of mitoKeima staining exhibiting primary hepatocyte mitophagy levels. Mean ± SEM, sample size (*n*) is indicated within the closed circle on each bar, *p < 0.05 vs. scrCK2α‐scrFUNDC1 group, #*p* < 0.05 vs. scrCK2α‐scrFUNDC1‐PA group, †*p* < 0.05 vs. siCK2α‐PA group.

**FIGURE 8:**
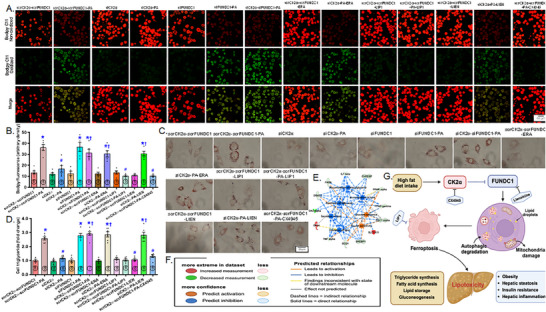
Effect of CK2α and FUNDC1 knockdown as well as CK2α selective inhibitor CX4945 (Silmitasertib) on palmitic acid (PA)‐induced changes in lipid peroxidation and steatosis in primary murine hepatocytes and HepG2 cells, respectively. Primary murine hepatocytes and HepG2 cells were transfected with siCK2α or siFUNDC1 prior to exposure to PA (0.5 mM) in the presence or absence of the ferroptosis inducer erastin (ERA, 20 µM), the ferroptosis inhibitor LIP1 (200 nM) or the mitophagy inhibitor liensinine (LIEN, 20 µM) for 72 hrs. A subset of PA‐treated HepG2 cells were incubated with the CK2α selective inhibitor CX4945 (10 µM). Scramble (scr) RNA serves as the negative control. A: Representative Bodipy C11 fluorescence images depicting lipid peroxidation from indicated primary hepatocyte groups; B: Quantification of Bodipy C11 fluorescence exhibiting lipid peroxidation in primary murine hepatocytes; C: Representative oil red O staining images from indicated HepG2 groups; D: Quantification of oil red O exhibiting intracellular triglyceride levels in HepG2 cells; E‐F: Disease and function analysis of CK2α knockout versus WT mice under HFD. This network illustrates differentially expressed proteins identified in this study. Red Nodes: Upregulated proteins; Green Nodes: Downregulated proteins. Orange Inner Ring: Predicted activation of associated functions; Blue Inner Ring: Predicted inhibition of associated functions. The box inset in panel F offers a denotations for these predictive indicators; and G: Schematic diagram exhibiting possible mechanisms for the role of CK2α and FUNDC1 in high fat diet‐induced hepatic steatosis. Long‐term high fat diet insult dampened FUNDC1‐mediated mitophagy through upregulation of CK2α, prompting disturbed lipid metabolism and lipid accumulation due to compromised mitophagy. Lipotoxicity then promotes onset of lipotoxic ferroptosis, imperiling hepatic steatosis. Created in BioRender. Lin, L. (2025) https://BioRender.com/i28p846. Mean ± SEM, sample size (*n*) is indicated within the closed circle on each bar, *p < 0.05 vs. scrCK2α‐scrFUNDC1 group, #*p* < 0.05 vs. scrCK2α‐scrFUNDC1‐PA group, †*p* < 0.05 vs. siCK2α‐PA group.

To decipher the possible role of mitophagy and ferroptosis in HFD‐induced changes in hepatic steatosis, we transfected both HepG2 cells and primary murine hepatocytes using siRNA targeting CK2α and FUNDC1 (scramble RNA serving as the control) before subjecting the cells to a 72‐hr palmitic acid (100 µM) challenge, with or without mitophagy suppressor liensinine, ferroptosis blocker liproxstatin‐1 (LIP1), or ferroptosis activator erastin. Mitophagy function, lipid peroxidation, and lipid buildup were evaluated using mitoKeima, Bodipy C11 fluorescence, and Oil Red O staining, respectively. The results demonstrated that palmitic acid suppressed mitophagy function in both HepG2 cells and primary hepatocytes, the responses were reversed by CK2α silencing. Interestingly, silencing of FUNDC1 negated the protective effect of siCK2α against palmitic acid‐evoked mitophagy defect in both HepG2 cells and primary hepatocytes, with minimal effects under control or palmitic acid challenge conditions (Figure 7A–D). Similarly, CK2α silencing reconciled palmitic acid‐evoked lipid peroxidation in primary hepatocytes and lipid buildup in HepG2 cells. Interestingly, silencing of FUNDC1 negated the beneficial response of siCK2α against palmitic acid‐instigated Bodipy C11 lipid peroxidation and lipid buildup in primary hepatocytes and HepG2 cells, respectively, with minimal effects observed under either control or palmitic acid challenge (Figure 8A–D). Induction of ferroptosis using erastin and inhibition of mitophagy using liensinine mitigated the beneficial effect of siCK2α against palmitic acid‐induced changes in mitoKeima intensity, Bodipy C11 fluorescence and lipid buildup, although neither treatment showed a notable effect in the absence of palmitic acid. Involvement of CK2α and ferroptosis in palmitic acid‐instigated mitophagy deficit, Bodipy C11 lipid peroxidation, and steatosis was further confirmed by the protective effect of the CK2α inhibitor CX4945 and the ferroptosis inhibitor LIP1 against palmitic acid insult (Figures 7A–D and 8A–D). Next, we performed disease and function analysis comparing CK2α^‐/‐^ versus the WTgroup under HFD condition. We quantified expression levels of 20 key proteins of our Western blot data [Figure 6 and Figure S1 (for supporting information) of our current study]. These quantitative protein expression data were then inputted into ingenuity pathway analysis (IPA) software for disease and function analysis. The resulting network visualization revealed that CK2α deletion overtly impacts lipid metabolism pathways and glucose homeostasis. Specifically, disease and function analysis predicted the inhibition of several lipid‐related processes (blue clusters), including lipid accumulation, triacylglycerol accumulation, fatty acid synthesis, lipid secretion, and lipid oxidation. Simultaneously, IPA predicted activation of glucose tolerance and insulin sensitivity (shown as orange clusters). The network identified altered expression of key regulatory factors, with elevated expression shown in red (such as SLC7A11, GPx4) and decreased expression shown in green (including CK2α, TNFα). These findings suggest that CK2α knockout may correct glucose and lipid metabolic disorders under a high‐fat diet (Figure 8E,F).

## 3 Discussion

Clinical and experimental evidence highlights the beneficial role of coordinated mitophagy and mitochondrial integrity in hepatocellular carcinoma, hepatic steatosis, and hepatitis [9, 30]. However, the role of mitophagy in MASLD remains poorly understood. Salient findings from our work include: (1) CK2α is upregulated, while FUNDC1 is downregulated in livers from MASLD patients, HFD‐fed mice, and palmitic acid‐treated hepatocytes; (2) Hepatic CK2α deletion alleviates HFD‐induced obesity, global metabolic derangement, liver injury, mitochondrial damage, suppressed mitophagy, ferroptosis, and aberrant lipid metabolism; (3) The protective effects of CK2α deletion against HFD‐induced liver damage require FUNDC1, as FUNDC1 deficiency nullifies these benefits; and (4) CK2α disrupts FUNDC1 homeostasis, compromising mitochondrial integrity, and promoting lipid peroxidation, ferroptosis and MASLD development (Figure 8G). This work should represent the first study to elucidate the CK2α‐FUNDC1‐mediated pathogenesis of MASLD possibly associated with the regulation of mitochondrial integrity and ferroptosis.

HFD consumption induces liver steatosis, fibrosis, and hepatomegaly, accompanied by elevated serum ALT, AST, cholesterol, and triglycerides. CK2α knockout mitigates these effects, reduces HFD‐induced obesity, and preserves hepatic morphology and lipid profiles, denoting an etiological role for CK2α in the hepatic lipid accumulation and liver damage in response to high‐fat diet intake. Whole‐body metabolic analysis (CLAMS) fails to support a crucial role for food intake and energy expenditure in CK2α ablation‐offered hepatic benefit, although CK2α ablation shifts energy utilization back to carbohydrate oxidation, manifested as improved RER and CO_2_ production. Following HFD intake, RER, and CO_2_ production were overtly decreased, denoting an abrupt switch in the energy source from carbohydrate to fat oxidation [31], the effect was reversed by CK2α knockout. CK2α deletion also improves glucose and insulin sensitivity [intraperitoneal glucose tolerance test (IPGTT), plasma insulin, and HOMA‐IR index] while reversing the upregulation of lipid synthesis and storage enzymes, favoring preserved glucose and lipid metabolism. Sustained lipid buildup would transit the mitochondrial metabolism in favor of ß‐oxidation, resulting in ROS production and mitochondrial damage, en route to hepatocyte death [32]. These findings suggest that CK2α contributes to HFD‐induced hepatic lipid accumulation and mitochondrial damage.

Our findings reveal that HFD upregulates hepatic CK2α, TNFα, IL1β, and markers of apoptosis (TUNEL, caspase‐3), dampens mitophagy (TOM20, FUNDC1), and promotes ferroptosis [Fe^2+^, malondialdehyde (MDA), GPX4, SLC7A11]. CK2α deletion reverses these changes, except for NCOA4, indicating an obligatory role for mitophagy (unlikely ferritinophagy) and ferroptosis in CK2α‐regulated hepatic injury. Earlier findings from our lab and others have denoted vital involvement for compromised autophagy, mitophagy, and proinflammatory response in hepatic steatosis and liver injury [33, 34]. Of note, mitophagy is known to alleviate lipid buildup via suppression of de novo lipogenetic genes encompassing SREBP1c, FAS, acetyl‐CoA carboxylase (ACC), and PPARα in HFD‐exposed livers [35, 36]. Defective mitophagy, including CK2α‐driven phosphorylation of FUNDC1 [22], exacerbates mitochondrial damage, and oxidative stress, thus fostering MASLD progression. Restoring FUNDC1 mitophagy in CK2α knockout mice helps clear excessive lipid droplets, underscoring the protective role of FUNDC1 in mitochondrial homeostasis. This is in line with the fact that FUNDC1 removal nullified CK2α knockout‐elicited benefit against HFD‐evoked hepatic steatosis and injury. Our study noted upregulation of the proinflammatory response (TNFα and IL1β) and the proinflammatory molecule CK2α following HFD intake, a response that was reverted by CK2α knockout. These results further favor the contribution of inflammation in CK2α‐facilitated hepatic injury and CK2α ablation‐elicited intervention of liver injury and MASLD progression.

In our current study, HFD provoked mitochondrial damage, reconfirming the notion of mitochondrial protection in the management of MASLD. Under mitochondrial damage, cells employed orchestrated mitophagy to clear away long‐lived or damaged mitochondria to sustain mitochondrial homeostasis [14]. Mitophagy participates in lipid droplet clearance, with defective mitophagy associated with mitochondrial damage, lipid accumulation, and inflammation [37, 38]. Perturbation in mitochondrial repair machinery (such as under HFD intake) compromises cellular energy production and promotes cell death (apoptosis and ferroptosis), facilitating MASLD progression. Our findings of upregulated CK2α correlate with defective mitophagy (where CK2α phosphorylates FUNDC1 at Ser [13] to inactivate mitophagy [22]) in MASLD patients and HFD‐fed mice. CK2α‐FUNDC1 double knockout mice fail to replicate the protective effects of CK2α deletion, highlighting FUNDC1's critical role in mitigating HFD‐induced liver injury. FUNDC1 was reported to rescue against carcinogen diethylnitrosamine‐provoked hepatocarcinogenesis through the blockade of inflammasomes [24]. This is supported by the notion that FUNDC1 maintains cross‐talk between skeletal muscles and fats to combat diet‐induced obesity [39], underscoring an intrinsic role for the mitophagy receptor in the governance of mitochondrial integrity and cell homeostasis. Changes in in vivo protein markers of mitophagy also received compelling support from the in vitro mitoKeima experiment using both HepG2 cells and primary murine hepatocytes, where FUNDC1 silencing or mitophagy inhibition using liensinine mitigated siCK2α‐offered benefit against palmitic acid‐evoked dysregulation of mitophagy. Likewise, inhibition of mitophagy using liensinine or induction of ferroptosis using erastin removed siCK2α‐evoked protection against palmitic acid‐induced lipid peroxidation and steatosis in primary murine hepatocytes and HepG2 cells, highlighting an upstream role for mitophagy in lipid peroxidation‐associated hepatic steatosis. Taken together, our findings offer the first evidence of a CK2α‐FUNDC1‐ferroptosis axis in MASLD and support targeting this pathway to enhance mitochondrial integrity, and combat ferroptosis, and MASLD progression.

Experimental limitations: Our study suffers from several limitations. First, the use of global knockout models may not fully recapitulate tissue‐specific effects, particularly within hepatocytes versus non‐parenchymal liver cells. The hepatic benefit offered by CK2α deletion against HFD may possibly be a secondary response to its global metabolic responses. Second, while murine models and in vitro hepatocytes (HepG2 and primary murine hepatocytes) were employed, these may not entirely capture human pathophysiology, warranting further validation in humanized or patient‐derived models. Third, although mitophagy and ferroptosis were implicated in HFD‐induced hepatic injury, the precise signaling cascades linking CK2α‐FUNDC1 interaction to downstream lipid and redox signaling remain incompletely defined. Last but not least, while our current study indicated the utility of CK2α inhibition, the long‐term safety and pharmacokinetics of CK2α‐targeted therapies in MASLD require further investigation.

In summary, our findings indicate that genetic ablation of CK2α restores FUNDC1 mitophagy, and alleviates mitochondrial injury and ferroptosis to protect against HFD‐induced hepatic metabolic injury and MASLD progression (Figure 8G). The obligatory role of FUNDC1 in the hepatic protection of CK2α deletion receives support from the fact that CK2α‐FUNDC1 double deficient mice were unable to recapitulate protection against HFD‐associated hepatic damage. Our results also demonstrated that selective small‐molecule CK2α inhibitor CX4945 (Silmitasertib, through competitive coupling to ATP‐binding site of CK2α) recapitulated CK2α silencing‐evoked changes in mitoKeima mitophagy, lipid peroxidation, and lipid droplet accumulation. CX‐4945 represents the first CK2 inhibitor to enter clinical trials with applications in various cancers, such as chronic lymphocytic leukemia, cholangiocarcinoma, and basal cell carcinoma [40, 41]. A number of clinical trials are ongoing using CX‐4945 in kidney cancer (NCT03571438), cholangiocarcinoma (NCT02128282), medulloblastoma (NCT03904862), and basal cell carcinoma (NCT03897036), although little is known for the impact of CK2α inhibition in MASFLD. Findings from our current study suggest promises of targeting CK2α in alleviating MASFLD‐associated liver damage possibly associated with the governance of FUNDC1, mitochondrial integrity, and ferroptosis. Nonetheless, CK2α may interact with drug‐metabolizing enzymes, thus affecting the pharmacokinetics of certain medications to impact drug efficacy or toxicity, particularly in patients with metabolic disorders. Future studies should explore the impact of CK2α manipulation on drug metabolism and interactions, particularly in the context of MASLD, to better understand how targeting CK2α might influence the treatment of MASLD and related conditions. Moreover, while the current study emphasizes the protective effects of CK2α inhibition in MASLD‐induced liver injury, the findings could have broader implications for other organs impacted by high fat intake, such as the kidneys, lungs, and heart. Further study is warranted to delineate these systemic responses and evaluate the potential of CK2α inhibition as a therapeutic strategy for metabolic diseases affecting multiple organs.

## 4 Materials and Methods

### 4.1 CK2α and FUNDC1 Knockout Mice, Generation of CK2α‐FUNDC1 Double Knockout Mice, and HFD Intake

The animal experiments received approval by the Animal Care and Use Committees (IACUC) at the Zhongshan Hospital Fudan University (Shanghai, China, No. 20220530) and Guangdong Second Provincial General Hospital (Guangzhou, China, No. 20200708‐BSGZ‐06‐01). In brief, global CK2α knockout (CK2α–^/–^) and FUNDC1 knockout (FUNDC1–^/–^) mice were generated and characterized in our laboratory as previously reported [22, 42, 43]. CK2α–^/–^ and FUNDC1–^/–^ mice, both on a C57BL/6 background, were bred for at least three generations to produce CK2α–^/—^‐FUNDC1–^/–^ double knockout mice. Genotyping was performed using PCR with the following primers: FUNDC1 5′‐AGACACCACTGGTGGAATCGAG (F), 5′‐CCTTCTGGAATAAAAATCCTGCAC (R); CK2α: 5′‐CCGCTTCCACCACAGTTTGA (F), and 5′‐TAAACTCTGGCCCTGCTTGG (R). Mice were maintained at 22 ± 2°C with 55 ± 5% humidity, with a 12 h/12 h light–dark circadian cycle and access to food and water ad libitum. Five week‐old male CK2α–^/–^, FUNDC1–^/–^ knockout, CK2α–^/—^‐FUNDC1–^/–^ double knockout and their wild–type littermates received either a low fat (Catalog # D12450B, 10% fat calories, Research Diets, New Brunswick, NJ, USA) or high fat (Catalog # D12492, 60% fat calories) diet for 20 weeks [44, 45]. To minimize gender‐related variability, male mice were utilized for our study. Sample sizes were determined through power analysis to ensure statistical robustness.

### 4.2 Bioinformatics Microarray Analysis

The raw data from GSE48452 was downloaded from the Gene Expression Omnibus (GEO) database (www.ncbi.nlm.nih.gov/geo/) [46] and included liver samples categorized as control (14 individuals), healthy obese (27 individuals), steatosis (MASLD, 14 individuals), and MASH (18 individuals), for mRNA profiling [47]. Microarray data processing was conducted using the “Oligo” package from R software [48]. RMA algorithm was applied for probe summarization and normalization, integrating probe intensities to obtain corrected data while minimizing background noise and errors [49]. Invalid probes were removed, and for genes with multiple probes, the median expression levels were calculated. DEGs were gathered with the assistance of the “limma” package, using |log2 fold change (FC)| > 1.0 and an adjusted *p* value < 0.05 [Benjamini & Hochberg (B‐H) method] [49, 50]. Clinical information for all samples was retrieved from the original paper of the GEO database, enabling the feature‐gene association analysis [47]. Kyoto Encyclopedia of Genes and Genomes (KEGG) and GO enrichment analyses of the identified DEGs were performed using the “clusterProfiler” package [51]. Genes were categorized by GO annotations into three domains: CC, MF, and BP. In addition, the “fgsea” and “GSVA” packages facilitated GSVA [52] and GSEA [53], respectively, identifying enriched gene sets and biological pathways, generating enrichment scores, and comparing gene expression patterns. Gene set activity scores were computed to unveil variation patterns, emphasizing the importance of these gene sets in BP. Significant pathways were identified with a B‐H adjusted *p* value < 0.05.

For PCA and partial correlation assessments, partial correlation coefficients were calculated between variables while controlling for confounders. The statistical significance of these coefficients was tested to confirm correlations between pathways and disease progression. A correlation matrix was constructed from the partial correlation coefficients, visualized in a structured plot illustrating interactions between enriched pathways and clinical features [54, 55]. PCA was performed on key enriched pathway scores, standardizing gene set enrichment scores and projecting data onto principal components to reduce dimensionality. Scores for each sample were calculated on the principal components, and characteristic vectors were analyzed to interpret the component's biological significance [56, 57]. Three principal components were selected as coordinate axes in a three‐dimensional space. Using the “scatterplot3d” package, a three‐dimensional plot was generated with varying colors or sizes of points to distinguish different sample clusters [56, 57].

### 4.3 Human Samples

Liver samples were obtained from transplant donors diagnosed with MASLD and non‐MASLD (anthropometric details provided in Table S1, for supporting information), in accordance with a protocol approved by the Ethics Committee of Zhongshan Hospital Fudan University (approval number: B2020–127R). All procedures were performed in compliance with the ethical principles of the Declaration of Helsinki for research involving human subjects.

### 4.4 Serum Levels of Lipid and Insulin, IPGTT

Serum triglycerides, insulin, and cholesterol levels were evaluated using commercial kits (EMD Millipore Corp, Billerica, MA, and BioVision, Inc. Mountain View, CA, USA) [45]. Serum levels of AST and ALT were assessed using Biovision colorimetric assay kits [58]. For IPGTT analysis, mice were fasted for 12 h prior to receiving a glucose challenge (2 g/kg body weight, i.p.). Blood glucose was determined at the base (0 min) and at 15, 60, and 120 min post‐injection using an Accu‐Chek III glucometer. AUC was determined with the assistance of trapezoidal method [45].

### 4.5 Metabolic Assessment

Each animal was individually housed in a metabolic cage with ad libitum access to food and water, using a Comprehensive Laboratory Animal Monitoring System (CLAMS, Columbus Instruments, Columbus, OH, USA) to assess metabolic parameters. The indices measured included oxygen consumption (VO_2_), carbon dioxide production (VCO_2_), RER (derived from VCO_2_/VO_2_), physical activity, and heat production [(3.815 + 1.232 × RER) × VO_2_) × 1000] [34].

### 4.6 Hepatic Triglycerides

Liver triglyceride was evaluated with a Biovision kit. Liver tissues were collected following animal sacrifice and heated (90°C) in a 5% NP‐40 buffer for 5 min. The solubilized triglycerides were spun, and the supernatant was used for triglyceride quantification with the assistance of a spectrophotometer [34].

### 4.7 Tissue Histology, Oil Red O Staining

Liver, white adipose, and brown adipose samples were fixed in 10% neutral‐buffered formalin for 24 h, followed by paraffin embedding. Tissues were sectioned into 7‐µm slices and subjected to hematoxylin and eosin (H&E), Masson trichrome, and Oil Red O staining. The extent of interstitial fibrosis was quantified by measuring the area stained blue (Masson's trichrome) as a proportion of the total microscopic field [34].

### 4.8 Electron Microscopy

Left ventricular tissues (1 mm^3^) were fixed in 2.5% glutaraldehyde and 1% osmium tetraoxide, followed by washing with a phosphoric acid buffer and dehydration in graded alcohol. The samples were embedded in Epon resin, sectioned using an ultramicrotome, and subsequently double‐stained with lead citrate and uranyl acetate. Ultrastructural images were acquired with a transmission electron microscope (FEI Tecnai G2 12) [59].

### 4.9 Mitochondrial Aconitase

Aconitase function was measured using the Aconitase‐340 assay kit (OxisResearch, Portland, OR, USA). Tissues were prepared in a 96‐well plate containing isocitrate dehydrogenase (enzyme), trisodium citrate (substrate), and NADP (50 µL each), and maintained for 15 min followed by absorbance reading at 340 nm [60].

### 4.10 Assessment of Superoxide (O_2_^−^)

Tissue slices were treated with 1 µM dihydroethidium (DHE, Molecular Probes, Eugene, OR, USA) at 37°C for half an hour, followed by washing and fluorescence measurement using a microplate reader [59].

### 4.11 TUNEL Staining

Paraffin‐embedded tissue slices were treated with Proteinase K buffer, followed by incubation with a reaction mixture containing terminal deoxynucleotidyl transferase (TdT), fluorescein‐dUTP. DNA strand breaks were detected by identifying TUNEL‐positive nuclei using a fluorescence detection kit (Roche, Indianapolis, IN, USA). Fluorescence images were acquired using an Olympus fluorescence microscope [61]

### 4.12 Determination of Tissue Fe^2+^ and MDA Content

Approximately 0.1 g of liver tissues were homogenized, and supernatants were gathered following centrifugation (4000 × *g* for 10 min). For Fe^2+^ content measurement, the supernatants were prewarmed for 30 min and absorbance was measured at 520 nm using an iron detection kit (Beijing Solarbio Science & Technology Co., Ltd., catalog number: BC4355) [62]. MDA levels in liver tissues were quantified using a kit from the Nanjing Jiancheng Institute of Biological Engineering [59].

### 4.13 Western Blot Analysis

Following homogenization, tissue samples were spun at 13,000 × *g* for 20 min. An equal amount of proteins (30 µg per sample) were separated by SDS–polyacrylamide gel electrophoresis using a Bio‐Rad gel system and transferred onto nitrocellulose membranes. Membranes were then blocked and maintained overnight at 4°C with primary antibodies targeting the following proteins: FAS, ACC, phospho‐ACC (Ser^79^), CHREBP, SREBP1c, PGC1α, PPARα, PPARγ, SCD1, DGAT1, PEPCK, G6Pase, IL1β, TNFα, TOM20, FUNDC1, α‐tubulin and GAPDH (as loading controls). Antibodies were obtained from Abcam (Cambridge, MA, USA) or Cell Signaling Technology (Danvers, MA, USA). After washing, the membranes were incubated with horseradish peroxidase (HRP)‐conjugated secondary antibodies (1:5,000 dilution) for 1 h. Gel bands were imaged using chemiluminescence and quantified by densitometric analysis with Quantity One software (Bio‐Rad) [34].

### 4.14 Primary Murine Hepatocyte Isolation

Adult C57BL/6J mice (4‐month‐old) were sedated using xylazine (12 mg/kg, i.p.) and ketamine (80 mg/kg). Portal veins were cannulated and perfused with an EGTA/Krebs‐Ringer solution for 10 min at 37°C. Hepatocytes were enzymatically digested using a calcium‐supplemented Krebs‐Ringer buffer containing Liberase (Roche) at 37°C for 10 min. The resulting cell suspension was filtered through a gauze and resuspended in a plating medium composed of 10% FBS, 2 mM glutamine, 200 nM dexamethasone, and penicillin‐streptomycin. A total of 3 × 10^5^ cells hepatocytes were seeded onto collagen‐coated culture plates and allowed to recover overnight prior to experimental procedures [63].

### 4.15 Mitophagy Assessment Using mitoKeima

Mitophagy was assessed using mt‐Keima (Hanheng Technology), a lysosomal protease‐resistant pH‐sensitive dye. Upon mitochondrial delivery to the acidic lysosomal environment, the probe's excitation peak shifts from 457 nm (emitting green fluorescence) to 561 nm (red fluorescence). HepG2 cells or primary murine hepatocytes were transfected with mitoKeima adenoviruses (multiplicity of infection = 20) for 48 h. Fluorescence captured under a laser confocal microscope (Leica) was analyzed using the ImageJ software [64].

### 4.16 Lipid Peroxidation

Lipid peroxidation in primary murine hepatocytes was assessed using BODIPY 581/591 C11 (D3861, Invitrogen), a fluorescent probe with a maximal absorption peak at 647 nm (red) and 594 nm (green). Fluorescence signals were detected using a Leica confocal microscope (TCS SP8, STED3X) [65].

### 4.17 Lipid and Triglyceride Levels

Lipid content was assessed using Oil Red O staining. HepG2 cells treated with various reagents were fixed in 10% formalin and incubated with Oil Red O for 20 min. Cells were then captured under light microscopy (400 ×), while the Oil Red O intensity was quantitated using a spectrophotometer at 530 nm [66, 67].

### 4.18 Ingenuity Pathway Analysis

Mouse tissue samples from four groups (WT‐LFD, WT‐HFD, CK2α^‐/–^LFD, and CK2α^‐/–^HFD) underwent Disease and Function annotation using IPA, (Qiagen, USA, https://digitalinsights.qiagen.com/IPA). Expression data for 20 molecules, validated through western blotting, were uploaded into IPA. A core analysis was performed, mapping the data onto IPA's extensive knowledge base. The Disease and Function analysis aimed to identify affected cellular processes and potential disease associations linked to the observed gene expression changes.

### 4.19 Data Analysis

Experimental data were presented as Mean ± SEM. A *p*‐value < 0.05 was used to determine statistical significance using analysis of variance (ANOVA) followed by Tukey's *post hoc* test. The Shapiro–Wilk test was employed to assess the normality of data distribution.

## Author Contributions

K.H., H.Z., Liheng L, F.L. and J.R. were involved in conception, design, and manuscript preparation. K.H., M.Z., X.L., R.L., F.L., J.L., Liheng L, Ling L, Y.W., H.C. performed the experiments, data analysis and interpretation; G.D.L. was involved in manuscript editing. All authors have read and approved the final manuscript.

## Ethics Statement

Animal study approval: Zhongshan Hospital Fudan University (Shanghai, China, No. 20220530) and Guangdong Second Provincial General Hospital (Guangzhou, China, No. 20200708‐BSGZ‐06‐01). Human study approval: Zhongshan Hospital Fudan University (approval number: B2020–127R).

## Conflicts of Interest

The authors declare no conflicts of interest.

## Supporting information




**Supporting File 1**: mco270277‐sup‐0001‐SuppMat.docx

## Data Availability

All data associated with this work are included here (main tex or supplementary files) and will be provided available upon reasonable request to the corresponding authors.
