# Supplementary material for: Casein Kinase 2α Ablation Confers Protection Against Metabolic Dysfunction‐Associated Steatotic Liver Disease: Role of FUN14 Domain Containing 1‐Dependent Regulation of Mitophagy and Ferroptosis
Source: MedComm (2020). 2025 Jul 11;6(7):e70277. doi: 10.1002/mco2.70277 (PMC12246556; doi:10.1002/mco2.70277)
Supplement: Supplementary file 1 — Supporting File 1: mco270277‐sup‐0001‐SuppMat.docx [file MCO2-6-e70277-s001.docx]

**Supplemental Materials**

**Casein Kinase 2α Ablation Confers Protection against Metabolic Dysfunction-Associated Steatotic Liver Disease: Role of FUN14 Domain Containing 1-Dependent Regulation of Mitophagy and Ferroptosis**

**Ke He^1#^, Meixiao Zhan^2,3#^, Xuanming Luo^4,5#^, Ruibing Li^6#^, Ling Lin^7,8^, Jie Lin^7,8^, Liheng Li^1^, Hongdong Chen^1^, Gary D. Lopaschuk^9^, Hao Zhou^10^, Fei Liu^1^*, and Jun Ren^7,8^***

**^1^Minimally Invasive Tumor Therapies Center, Guangdong Second Provincial General Hospital, Guangzhou, Guangdong 510317, China; ^2^Guangzhou First People's Hospital, the Second Affiliated Hospital, School of Medicine, South China University of Technology, 510001, China; ^3^Guangdong Provincial Key Laboratory of Tumor Interventional Diagnosis and Treatment, Zhuhai People’s Hospital (Zhuhai Hospital Affiliated with Jinan University), Zhuhai, Guangdong 519000, China; ^4^Department of Biliary Tract Surgery, Zhongshan Hospital Fudan University 200032, China; ^5^Department of General Surgery, Shanghai Xuhui Central Hospital Fudan University, Shanghai, 200031, China; ^6^Department of Clinical Laboratory Medicine, The First Medical Centre, Medical School of Chinese People’s Liberation Army, Beijing 100037, China; ^7^Department of Cardiology, Shanghai Institute of Cardiovascular Diseases, Zhongshan Hospital Fudan University, Shanghai 200032, China;** **^8^National Clinical Research Center for Interventional Medicine, Shanghai, 200032, China; ^9^Cardiovascular Research Centre, University of Alberta, Edmonton, Alberta T6G 2S2 Canada; ^10^Senior Department of Cardiology, The Sixth Medical Center of People’s Liberation Army General Hospital, Beijing 100048 Beijing, China**

***Equal contribution**

**Running title: CK2α imposes MASLD injury via mitophagy**

**Correspondence to: Dr. Jun Ren (E-mail: ren.jun@zs-hospital.sh.cn) or Dr. Fei Liu (E-mail:** [**kqliufei@126.com**](mailto:kqliufei@126.com)**)**

Supplementary Information Table S1: General anthropometric information of MASLD and non-MASLD participants

|  | Non-MASLD | MASLD |
| --- | --- | --- |
| All | 15 | 15 |
| Male Gender | 1 (6.7%) | 10 (66.7%) |
| Age (years) | 41.2 ± 2.9 | 29.5 ± 3.1* |
| ALT (U/L) | 82.3 ± 18.4 | 40.8 ± 5.3 |
| AST (U/L) | 32.9 ± 4.9 | 30.5 ± 4.0 |
| Steatosis Grade (0–3) | 0 | 2.0 ± 0.3* |

Mean ± SEM, * *P* < 0.05 vs. Non-MASLD group.

Supplementary Information Figure 1: Levels of CK2α, inflammatory, mitophagy and ferroptosis markers in livers from WT, CK2α knockout (CK2α^–/–^), FUNDC1 knockout (FUNDC1^–/–^) and CK2α-FUNDC1 double knockout (CK2α^–/–^-FUNDC1^–/–^) mice placed on a 60% high fat (HF) or nutritionally matched low fat (LF) diet for 20 weeks. A: Representative immunoblots depicting levels of CK2α, TNFα, IL1β, caspase3, TOM20, FUNDC1, GPx4, SLC7A11 and NCOA4 (GAPDH as loading control); B: CK2α levels; C: TNFα levels; D: IL1β levels; E: Caspase 3 (cleaved) levels; F: TOM20 levels; G: FUNDC1 levels; H: Hepatic tissue Fe^2+^ levels; I: Malonaldehyde levels; J: GPx4 levels; K: SLC7A11 levels and L: NCOA4 levels. Mean ± SEM, sample size (n) is indicated within the closed circle on each bar. *p < 0.05 *vs.* WT mice, #p < 0.05 *vs.* WT-HF mice, †p < 0.05 *vs.* CK2α^–/–^-HF mice.
